# Bcl2 Family Functions as Signaling Target in Nicotine-/NNK-Induced Survival of Human Lung Cancer Cells 

**DOI:** 10.1155/2014/215426

**Published:** 2014-05-20

**Authors:** Xingming Deng

**Affiliations:** Division of Cancer Biology, Department of Radiation Oncology, Emory University School of Medicine and Winship Cancer Institute of Emory University, Atlanta, GA 30322, USA

## Abstract

Lung cancer is the leading cause of cancer death and has a strong etiological association with cigarette smoking. Nicotine and nitrosamine 4-(methylnitrosamino)-1-(3-pyridyl)-1-butanone (NNK) are two major components in cigarette smoke that significantly contribute to the development of human lung cancer. Nicotine is able to stimulate survival of both normal human lung epithelial and lung cancer cells. In contrast to nicotine, NNK is a more potent carcinogen that not only induces single-strand DNA breaks and oxidative DNA damage but also stimulates survival and proliferation of normal lung epithelial and lung cancer cells. However, the molecular mechanism(s) by which nicotine and NNK promote cell survival, proliferation, and lung tumor development remains elusive. The fate of cells (i.e., survival or death) is largely decided by the Bcl2 family members. In the past several years, multiple signaling links between nicotine/NNK and Bcl2 family members have been identified that regulate survival and proliferation. This review provides a concise, systematic overview of the current understanding of the role of the pro- or antiapoptotic proteins in cigarette smoking, lung cancer development, and treatment resistance.

## 1. Introduction

The best currently available therapies for lung cancer patients achieve overall 5-year survival rates of 16% and 6% for NSCLC and SCLC, respectively [1]. It has been demonstrated that lung cancer has a strong etiological association with cigarette smoking [2, 3]. Nicotine and nitrosamine 4-(methylnitrosamino)-1-(3-pyridyl)-1-butanone (NNK) are two important components in cigarette smoke [4]. Recent reports indicate that nicotine promotes survival of both normal human lung epithelial and lung cancer cells [5, 6]. In contrast to nicotine, NNK is a more potent carcinogen that not only induces single-strand DNA breaks and oxidative DNA damage but also stimulates survival and proliferation of normal lung epithelial and lung cancer cells [6–8]. However, the molecular mechanism(s) by which nicotine and NNK promote cell survival, proliferation, and lung tumor development remains elusive. The fate of cells (i.e., survival or death) is largely decided by the Bcl2 family members [9, 10]. The subfamily members including Bcl2, Bcl-XL, and Mcl-1 inhibit apoptosis, whereas the Bax subfamily, consisting of Bax and Bak, as well as the BH3-only subfamily, including Bad, Bid, Bok, Bik, and Bim, promotes apoptosis [11–16]. The Bcl2 family members have homology clustered within four conserved Bcl2 homology (BH) domains: BH1, BH2, BH3, and BH4, in which only antiapoptotic proteins, such as Bcl2, Bcl-XL, Bcl-w, and A1, bear the NH_2_-terminal BH4 domain [17]. In contrast, Mcl-1 has a helical BH4-like domain which is located between the PEST region and the BH3 domain [18]. The proapoptotic family members can be divided into two subgroups based on the presence of BH domains: the BH123 multidomain proteins (i.e., Bax and Bak) and the BH3-only molecules [19–21]. Recent studies suggest that there are two different subgroups among the BH3-only members. One group, including Bid and Bim, can function both directly to bind and activate Bax and indirectly to counteract the inhibition of Bax or Bak by antiapoptotic members including Bcl2 and Bcl-XL. Other BH3-only proteins (i.e., Bad, Bik, Noxa, and PUMA) lack the ability to directly activate Bax but can oppose the action of antiapoptotic family members. Bcl2 and related antiapoptotic proteins block the progression of a death signal by preventing Bax/Bak oligomerization [22]. Importantly, we have recently discovered that the cigarette smoke components, nicotine and NNK, can induce phosphorylation of Bcl2, Mcl-1, Bax, and Bad, which leads to activation of Bcl2 and Mcl-1 [5, 23] and inactivation of Bax and Bad in association with increased chemoresistance in human lung cancer cells [24, 25]. Additionally, NNK simultaneously induces both Bcl2 phosphorylation and c-Myc phosphorylation, which promote functional cooperation of these two oncogenic proteins in lung cancer development.

## 2. Discussion

### 2.1. Cigarette Smoking and Human Lung Cancer

Cigarette smoking is by far the most important risk factor in the development of lung cancer [26]. Cigarette smokers have a 20-fold higher relative risk of developing lung cancer compared with nonsmokers, and 90% of all lung cancers are caused by smoking [27]. In the United States, cigarette smoking alone causes approximately 30% of cancer deaths and a total of 440,000 premature deaths annually, most from lung and other cancers. An estimated amount of $157 billion in annual health-related economic losses is contributable to smoking [28].

### 2.2. Nicotine, a Major Component in Tobacco, Activates Both Survival and Growth-Promoting Pathways to Facilitate the Development of Lung Cancer

Cigarette smoke contains about 4,000 chemicals, 55 of which have been evaluated as carcinogens [29]. Nicotine is a major component in tobacco that exists at high concentrations (90–1000 nM) in the blood of smokers [30]. High affinity nicotinic acetylcholine receptors (nAChRs) are found on human lung cancer and normal lung cells [31, 32]. Nicotine functions as a survival agonist to inhibit apoptosis induced by diverse stimuli including chemotherapeutic drugs [33]. However, the intracellular signal transduction mechanism(s) involved in nicotine suppression of apoptosis in human lung cancer cells remains enigmatic. Nicotine has been reported to activate a protein kinase cascade (i.e., PKC/Raf/MEK/ERKs) that may potentially promote survival and proliferation of human lung cancer cells [32–36]. It is interesting to identify the downstream survival and proliferative substrates of nicotine-activated protein kinases. ERK1 and ERK2 have been identified as physiological Bcl2 kinases [37], suggesting that nicotine should have the capacity to regulate Bcl2 to support survival and potentially promote lung cancer development.

### 2.3. Bcl2 Is a Potent Antiapoptotic Molecule and Its Phosphorylation in the Flexible Loop Domain Regulates Its Survival Function

The apoptotic process can be divided into three interdependent phases: induction, decision, and execution. The decision phase is largely regulated by the Bcl2 family of apoptotic regulators [10, 38]. Bcl2 can suppress cell death induced by a variety of stress applications including growth factor withdrawal, chemotherapy, irradiation, and viral infection [10, 38]. However, it is still not clear how Bcl2 actually functions to block apoptosis and promote survival. Bcl2 was initially identified as a potential phosphoprotein when expressed in SF9 insect cells where it was shown to prolong cell survival following baculovirus infection [39]. Recent reports indicate that the endogenous Bcl2 expressed in various cells can be phosphorylated and that phosphorylation of Bcl2 is closely associated with regulation of apoptosis [37, 40, 41]. We have discovered that phosphorylation of Bcl2 at S70 in the flexible loop domain (FLD) can positively regulate its antiapoptotic function [37, 42]. Since the nonphosphorylatable S70A Bcl2 mutant results in a significant loss of antiapoptotic function following various stresses, this indicates a survival role for the charge conferred by this posttranslational modification [42]. However, some residual antiapoptotic activities are associated with the S70A mutant [10, 37], suggesting that phosphorylation at other sites present in the FLD may also contribute to Bcl2's function. Indeed, Bcl2 can be phosphorylated at multiple sites in the FLD, including T69, S70, and S87, in association with inhibition of microtubule dynamics [41]. Importantly, conversion of S70 to glutamate (S70E), a charged amino acid that could potentially mimic phosphorylation at S70, resulted in increased cell survival [42]. These data strongly suggest that S70 is a regulatory site for Bcl2 and allowed us to conclude that phosphorylation at this site may be necessary for Bcl2's survival function [5, 42].

### 2.4. Multiple Protein Kinases Are Involved in Bcl2 Phosphorylation and Regulate Its Antiapoptotic Function

Bryostatin-1, a potent PKC activator, can induce Bcl2 phosphorylation at S70 [40, 42], suggesting that PKC appears to be a logical choice as a physiological Bcl2 kinase. We found that highly purified, activated PKC*α* directly phosphorylated Bcl2 exclusively at S70* in vitro* [43]. Furthermore, PKC*α* could be induced to translocate to the mitochondrial fraction following bryostatin addition to human pre-B REH leukemia-derived cells [43]. Overexpression of PKC*α* leads to increased Bcl2 phosphorylation and increased resistance to chemotherapy in REH cells [43]. These findings suggest that PKC*α* is a physiological Bcl2 kinase. However, high concentrations of staurosporine, up to 1 *μ*M, only partially inhibit IL-3 stimulated Bcl2 phosphorylation but completely block PKC-mediated Bcl2 phosphorylation* in vitro* [37], indicating a role for a staurosporine-resistant Bcl2 kinase (SRK). We have identified that ERK1/2 functions as a physiologic SRK that is able to induce Bcl2 phosphorylation at S70* in vitro *and* in vivo* [37]. As potential factors in lung cancer development, nicotine and NNK can activate both PKC and SRK (i.e., ERK1/2) physiological Bcl2 kinases [5, 37, 44] and inhibit chemotherapeutic drug-induced apoptosis in lung cancer cells [5, 29]. It is possible that nicotine- or NNK-induced inhibition of apoptosis may occur through phosphorylation of Bcl2.

### 2.5. Nicotine Induces Bcl2 Phosphorylation at S70 via Activation of PKC*α* and the ERK1/2 Protein Kinases, Leading to Enhanced Survival of Lung Cancer Cells

Nicotine is a survival agonist that inhibits apoptosis following various stresses [33], but the intracellular signal mechanism(s) that mediates this function remains unclear. Bcl2 is a cellular protooncogene that functions as a potent antiapoptotic molecule and tumor promoter [45]. High levels of Bcl2 are expressed in human lung cancer cells, while the level appears to be low in normal lung cells [46]. A report indicates a correlation between heavy cigarette smoking and increased expression of Bcl2 in patients with lung, head, and neck cancers, suggesting that Bcl2 may be a primary target of carcinogens in tobacco smoke [46]. In support of this, we found that high levels of endogenous Bcl2 are expressed in several lung cancer cell lines, including those from SCLC and NSCLC [47, 48]. Importantly, nicotine can stimulate phosphorylation of endogenous Bcl2 in SCLC H69 cells and enhances cell survival following treatment with chemotherapeutic drugs including VP16 and cisplatin [5]. Nicotine-induced Bcl2 phosphorylation occurs exclusively at the S70 site in association with prolonged survival of SCLC H82 cells expressing wild-type but not the phosphorylation-deficient S70A mutant Bcl2 after treatment with chemotherapeutic agents (i.e., cisplatin or VP-16) [5]. Importantly, nicotine induces Bcl2 phosphorylation through signaling pathways involving activation of PKC*α* and the MAPKs ERK1 and ERK2 in lung cancer cells. Since ET-18-OCH3, a specific phospholipase C (PLC) inhibitor, can block nicotine-stimulated Bcl2 phosphorylation and promotes apoptosis [5], it has been proposed that nicotine induces PLC activation that triggers the PKC/ERK1/2 kinase cascade to phosphorylate survival substrate and Bcl2 and promote cell survival [5]. Nicotine-induced cell survival results, at least in part, from a mechanism that involves Bcl2 phosphorylation at S70 [5]. Therefore, novel therapeutic strategies for lung cancer in which Bcl2 is expressed may be used to abrogate the antiapoptotic activity of Bcl2 by inhibiting multiple upstream nicotine-activated pathways.

### 2.6. Nicotine Induces Mcl-1 Phosphorylation in Association with Increased Survival of Human Lung Cancer Cells

Mcl-1 is a major antiapoptotic member of the Bcl2 family, which is extensively expressed in various human lung cancer cells [23, 49]. Mcl-1 is a unique member of the Bcl2 family because of its short half-life (30 minutes-3 hours in various cell types) and short-term prosurvival function, which probably relates to the presence of a long proline-, glutamic acid-, serine-, and threonine-rich (PEST) region upstream of the BH domain [50–53]. Thus, the mechanism(s) that prolongs the half-life of Mcl-1 protein is critical for its long-term survival function. Mcl-1 protein can be phosphorylated at multiple sites that distinctly regulate its protein turnover. For example, extracellular signal-regulated kinase (ERK)1/2-mediated phosphorylation at the Thr163 site enhances the half-life and antiapoptotic function of Mcl-1 [54, 55]. In contrast, S159 phosphorylation by GSK-3*β* facilitates Mcl-1 ubiquitination and degradation to reduce its survival activity [55]. Additionally, Cdk1/2-mediated phosphorylation at the S64 site increases the antiapoptotic function of Mcl-1 but has no effect on its half-life [50]. We recently discovered that nicotine promotes survival of human lung cancer cells through a novel mechanism by activating the antiapoptotic function of Mcl-1 via its phosphorylation [23].

Nicotine activates ERK1/2 through the upstream *β*-adrenergic receptor [56], which can induce Mcl-1 phosphorylation at the Thr163 site in the PEST region [23]. Nicotine-induced Mcl-1 phosphorylation at Thr163 enhances the half-life of Mcl-1, which leads to its long-term survival function and/or chemoresistance of human lung cancer cells [23]. Thus, disruption of the antiapoptotic function of Mcl-1 by blocking its Thr163 site phosphorylation may represent a new strategy for the treatment of tobacco-related cancer, especially for lung cancer and other malignancies that express Mcl-1.

### 2.7. Nicotine and NNK Inactivate the Proapoptotic Function of Bad through Phosphorylation

Bad is one of the BH3-only proapoptotic members, and phosphorylation of Bad at S^112^, S^136^, and S^155^ has been demonstrated to inactivate its proapoptotic function in a mechanism involving binding to 14-3-3 scaffold proteins which results in sequestration of Bad from mitochondria and dissociation of Bad from mitochondrial Bcl2 and/or Bcl-X_*L*_ [57–60]. The active Bad is a dephosphorylated form that localizes in the mitochondria and interacts with Bcl-X_*L*_ to neutralize its antiapoptotic function. ERKs, AKT, and PKA function as Bad S^112^, S^136^, and S^155^ kinases, respectively [61–64]. Nicotine has previously been demonstrated to potently activate both MAPKs ERK1/2 and AKT in association with increased survival of normal lung airway epithelial cells [6]. We discovered that nicotine potently induces Bad phosphorylation at S^112^, S^136^, and S^155^ in a mechanism involving activation of MAPKs ERK1/2, PI3 K/AKT, and PKA in human lung cancer cells [25]. Nicotine-induced multisite phosphorylation of Bad results in its sequestration from mitochondria and subsequent interaction with 14-3-3 in the cytosol. Interestingly, phosphorylation of Bad at S^112^ occurs earlier than at S^136^ or S^155^, suggesting that nicotine-induced multisite Bad phosphorylation may occur in a hierarchical manner [25]. Phosphorylation of S^112^ may facilitate further phosphorylation of Bad at S^136^ and S^155^ sites. Additionally, NNK has been found to stimulate multisite Bad phosphorylation at S^112^, S^136^, and S^155^ via activation of PKC*ι* in association with increased survival of human lung cancer cells [65]. Thus, in addition to Bcl2 and Mcl-1, nicotine- or NNK-induced survival may occur, at least in part through inactivation of the BH3-only molecule Bad by phosphorylation, which may contribute to the development of human lung cancer and/or chemoresistance.

### 2.8. Nicotine Negatively Regulates the Proapoptotic Function of Bax

Bax is a major proapoptotic protein whose activation is required for apoptotic cell death [66]. It has been reported that GM-CSF induces Bax phosphorylation at S184 in the hydrophobic C-terminal tail and inactivates the proapoptotic activity of Bax in neutrophils [67]. These findings reveal that the proapoptotic activity of Bax could be regulated by a posttranslational modification (i.e., phosphorylation). Because Bax is ubiquitously expressed in both SCLC and NSCLC cells, nicotine may mimic growth factor(s) to regulate the activity of Bax. As expected, nicotine has been found to induce Bax phosphorylation at S184, which results in abrogation of the proapoptotic activity of Bax and increased cell survival [24]. AKT, a known physiological Bax kinase, is activated by nicotine, colocalizes with Bax in the cytoplasm, and can directly phosphorylate Bax* in vitro*. Importantly, nicotine-induced Bax phosphorylation potently blocks stress-induced translocation of Bax from the cytosol to the mitochondria, impairs Bax insertion into mitochondrial membranes, and reduces the half-life of Bax protein [24]. Additionally, we identified PKC*ζ* as another nicotine-activated Bax kinase that is able to directly phosphorylate Bax in human lung cancer cells [68]. Therefore, nicotine-induced survival and chemoresistance of human lung cancer cells may occur through a mechanism involving activation of PI3 K/AKT and PKC*ζ* that directly phosphorylates Bax leading to inactivation of its proapoptotic function, which may contribute to the development and/or chemoresistance of human lung cancer.

### 2.9. NNK Promotes Functional Cooperation of Bcl2 and c-Myc through Phosphorylation in Human Lung Cancer Cells

Among the known protooncogenes, the cellular myc gene (c-Myc) is one of those most frequently implicated in carcinogenesis [69, 70]. Deregulated expression of the structurally unaltered Myc protein is sufficient to drive continuous cell proliferation and apoptosis in response to growth-promoting and growth-inhibitory signals, respectively [69]. Expression of the oncogene c-Myc can initiate proliferation and increase sensitivity to apoptosis under low serum conditions when antiapoptotic mechanisms are not activated [71]. Activation of the Raf/MEK/ERK and the PI3-K/AKT kinase cascades regulates the phosphorylation of two sites (i.e., Thr58 and S62) in the N terminus of c-Myc, which are conserved between all Myc family members and have opposing effects on Myc stability [72]. Since nicotine and NNK have been reported to induce activation of both the Raf/MEK/ERK and the PI3-K/AKT kinase cascades [6, 7, 44], these two distinct pathways may cooperate to regulate the stability of c-Myc through phosphorylation of S62 and Thr58. Bcl2 and c-Myc are two major oncogenic proteins that can functionally cooperate in cell proliferation, transformation, apoptosis, and tumorigenicity [73]. To avoid c-Myc-induced cell death and ensure continuous cell proliferation, Bcl2 functions as one of the most potent Myc-cooperating oncoproteins [74, 75], which is a global inhibitor of apoptosis, likelythrough multiple mechanisms [37, 69]. Bcl2 can specifically abrogate c-Myc-induced apoptosis without affecting the c-Myc mitogenic function [74]. Interestingly, NNK can simultaneously stimulate Bcl2 phosphorylation exclusively at S70 and c-Myc phosphorylation at Thr58 and S62 through activation of both ERK1/2 and PKC*α* [47], which facilitates a functional cooperation between Bcl2 and c-Myc leading to enhanced survival, proliferation, and chemoresistance of human lung cancer cells.

## 3. Conclusion 

**Figure 1:**
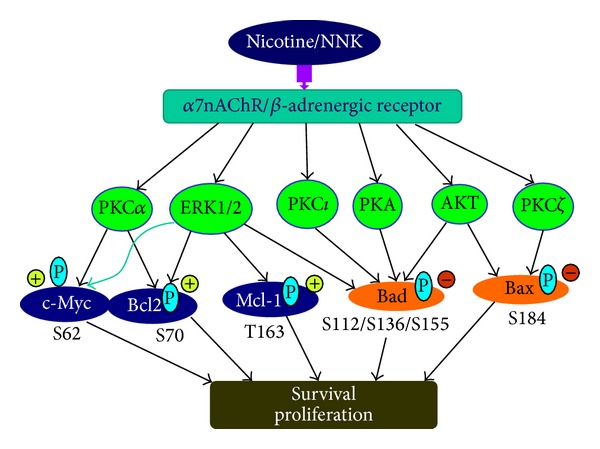
Proposed model of nicotine/NNK signaling in human lung cancer cells. Nicotine or NNK stimulates phosphorylation of Bcl2, Mcl-1, Bad, and Bax via activation of multiple protein kinases leading to activation of Bcl2/Mcl-1, inactivation of Bad/Bax, and promotion of functional cooperation between Bcl2 and c-Myc, which contributes to the survival and proliferation of human lung cancer cells.

Cigarette smoking is the most important risk factor in the development of lung cancer. Interestingly, cigarette smoke components (i.e., nicotine and NNK) can regulate Bcl2 family members through multiple signaling pathways in human lung cancer cells (Figure 1). Nicotine or NNK induces the phosphorylation of Bcl2, Mcl-1, Bad, and Bax through activation of ERK1/2, AKT, PKA, PKC*α*, PKC*ι*, and PKC*ζ*. Nicotine-/NNK-induced phosphorylation of Bcl2 and Mcl-1 enhances their antiapoptotic functions, while phosphorylation of Bax and Bad inactivates their proapoptotic functions, which contributes to increased survival and chemoresistance of human lung cancer cells. NNK can stimulate the functional cooperation of Bcl2 and c-Myc via phosphorylation, which may promote lung cancer development. To explore the relevance of the signaling pathways we have characterized, in cell lines for clinical patients, the phosphorylation status of Bcl2, Mcl-1, Bad, or Bax in tumor tissues from smoking and nonsmoking lung cancer patients which should be evaluated in future studies. Thus, our cell-based findings in combination with data from patients will provide strong clinical relevance for prognosis as well as for the treatment of tobacco-related cancers, specifically lung or other Bcl2 family and c-Myc expressing malignancies. These studies may contribute significantly to the development of novel strategies specifically aimed at functionally blocking multiple Bcl2 family signaling pathways.
